# Digital Stereotypes in HMI—The Influence of Feature Quantity Distribution in Deep Learning Models Training

**DOI:** 10.3390/s22186739

**Published:** 2022-09-06

**Authors:** Pawel Antonowicz, Michal Podpora, Joanna Rut

**Affiliations:** 1 Department of Computer Science, Opole University of Technology, Proszkowska 76, 45-758 Opole, Poland; 2 Faculty of Production Engineering and Logistics, Opole University of Technology, Sosnkowskiego 31, 45-272 Opole, Poland

**Keywords:** machine learning, feature measurement, cognitive technologies, artificial intelligence, Industry 5.0, digital stereotypes

## Abstract

This paper proposes a concept of Digital Stereotypes, observed during research on quantitative overrepresentation of one class over others, and its impact on the results of the training of Deep Learning models. The real-life observed data classes are rarely of the same size, and the intuition of presenting multiple examples of one class and then showing a few counterexamples may be very misleading in multimodal classification. Deep Learning models, when taught with overrepresentation, may produce incorrect inferring results, similar to stereotypes. The generic idea of stereotypes seems to be helpful for categorisation from the training point of view, but it has a negative influence on the inferring result. Authors evaluate a large dataset in various scenarios: overrepresentation of one or two classes, underrepresentation of some classes, and same-size (trimmed) classes. The presented research can be applied to any multiclassification applications, but it may be especially important in AI, where the classification, uncertainty and building new knowledge overlap. This paper presents specific ’decreases in accuracy’ observed within multiclassification of unleveled datasets. The ’decreases in accuracy’, named by the authors ’stereotypes’, can also bring an inspiring insight into other fields and applications, not only multimodal sentiment analysis.

## 1. Introduction

The idea of human-machine cooperation and partnership plays the key role in the current digital transformation—Industry 5.0—the transformation that goes beyond the horizon of intelligent factories and automated systems and processes. The fifth industrial revolution strongly incorporates cognitive technologies, making the system fabric more natural and native for human beings, enabling true coexistence and cooperation of machines and humans. The cognitive technologies are also increasingly often used in business, including within the communication process. Verbal interactions contain not only a message, but they also carry non-verbal clues, including emotional content, which can be processed using a multimodal sentiment analysis subsystem. A smile is an effective indicator in communication and in business communication, while at the same time, a smile is one of the simplest and most common face expressions.

Learning to distinguish one object class from another is one of the most basic cognitive tasks a human being is capable of. The same capability is being expected from artificial systems, machines or robots. However, the teaching-learning methodology is different for the above-mentioned cases, and the results also differ. Teaching a machine to acquire new knowledge does not always follow this path. Deep Learning is an example of a methodology of embedding knowledge within the layers of a Convolutional Neural Network, usually done in the learning stage, after which a model is obtained, which can be used for confronting new data with the learned concepts. On the other hand, learning new knowledge for a living being is done with reference to the current knowledge state, by showing similarities and differences, and it does not start with empty structures/layers. Some of the learning factors are quite intuitive (e.g., choosing images that clearly show the features that distinguish cats and dogs), but some of them tend to be omitted or forgotten (e.g., if considerable number of samples present cats sleeping on a red sofa, then the red sofa can become an indicator of a cat image). The paper’s objective is to reach the researchers who use deep networks (especially using the TensorFlow library) with the idea of examining one of the common (while often underrated) challenges in working with data—when some classes have more examples (instances within the teaching set) than other ones.

### 1.1. Motivation Background—The Importance of Smile in Business Relationships

The dynamics and the digitalization of economy and society and virtualization of sales channels and customer service channels [1,2], were reinforced during the pandemic [3]. Enhancing the unification of both virtual and real worlds, already back in the 2015, were said to have led to significant changes in marketing [4].

A smile is a basic component of the first impression. It also has an impact on building a relationship with the interlocutor/client. It is one of the most effective non-verbal communication techniques, and often says more than any spoken word. A smile can have many meanings: ’I’m glad to see you’, ’I like you’, ’I’m interested in you’. A smile also represents a satisfied customer, reaching an agreement in the implementation of business activities [5], joy, delight, or enthusiasm. People usually smile when they are happy, however, they also happen to smile when they’re unhappy, embarrassed, or even when they are offended. A smile can be used to transport (or disguise) various emotions and feelings, simple or complex, including: love, contempt, pride, delight, but also submission, awkwardness, mockery, or even disgust (although this might be detected [6]). According to [7], a human face is capable of expressing about 50 types of smiles, and each type can have a different meaning. Ref. [8] investigated the influence of smiles on the perception of verbal statements of positive, neutral, and negative meaning. The authors prove that smiling has shifted the perception of messages by making the negative ones appear less negative and neutral ones more positive. They also experimented with combining disgust and anger with smiling expression, and concluded that smiles are being used to moderate the meaning of verbal statements.

### 1.2. The Challenge

It is common knowledge for Deep Learning specialists that having more training (and testing) data is usually better, inter alia due to the overfitting reduction [9,10,11,12]. However there should also be a remark made, which is not equally often stated, that data distributed equally among the all classes in the dataset (instead of simply using as much data as it is possible regardless of the data distribution) also brings benefits. It must be noted that, while uniform data distribution among classes in this paper is presented as highly beneficial (from a classification point of view), it also introduces biases [13,14,15,16]. It should also be noted that, in many real-life use cases (e.g., the notion of balance of linguistic corpora [17,18,19]) a dataset that represents both classes with equal number of instances would not be representative (one class (word/phrase) can be represented with significantly less instances than other one).

An important and interesting question arises: how does the overrepresentation of one class affect the model and its prediction and generalization capability.

**Figure 1:**
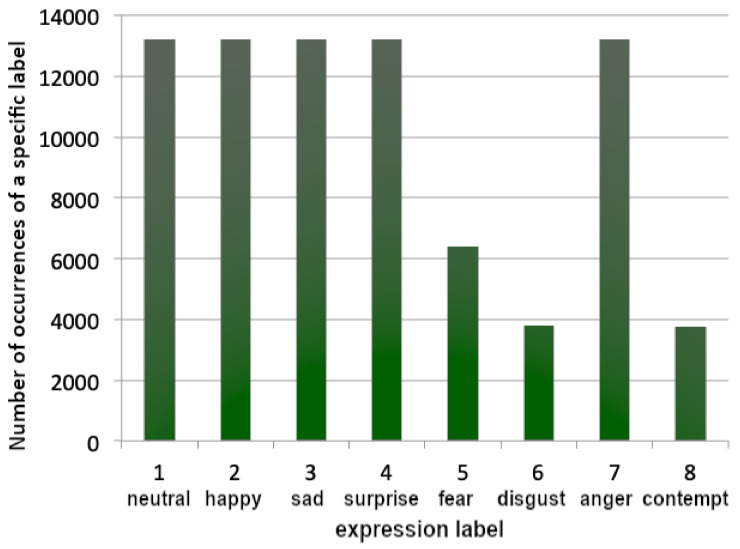
Data distribution of 80,000 images trimmed to avoid overrepresentation.

**Figure 2:**
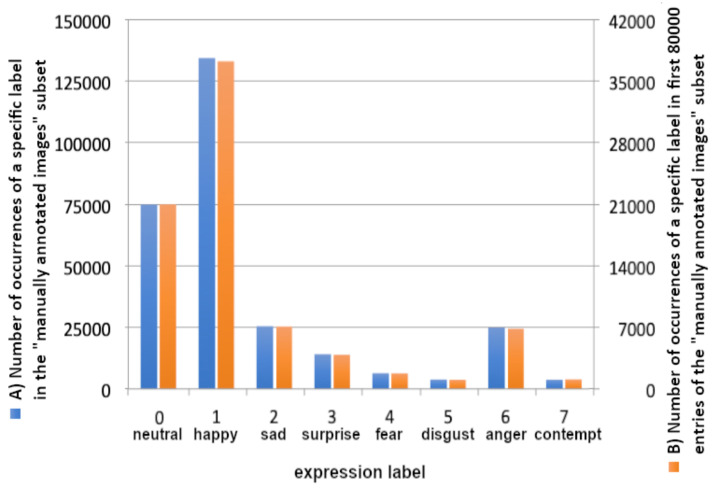
Training data distribution within the ’manually annotated images’ subset (A, left, blue) of the AffectNet [20] Dataset and within its first 80,000 records only (B, right, orange).

The authors performed over 100 trainings of models with varying training parameters using various training set sizes with both leveled and unleveled data distribution, intentionally not using data augmentation to study the influence of data distribution vs. data quantity. (By ’leveled’ authors mean avoiding overrepresentation by trimming the number of instances (Figure 1) as opposed to natural number of instances in a dataset (Figure 2)).

The analysis of the influence of the quantity of samples on the occurrence of the negative result (’stereotype’) within a non-binary classification using Deep Learning was performed using the AffectNet dataset [20] containing images of faces, labeled regarding face expressions.

## 2. Methodology

The AffectNet dataset [20] consists of two separate parts: manually annotated images and automatically annotated images. Both include 11 categories. Eight of them are for facial expressions (Neutral, Happy, Sad, Surprise, Fear, Disgust, Anger, Contempt), three are unrelated to facial expressions (None, Uncertain, Non-Face). While the targeted model is intended for facial expression recognition [21], thus the first eight (facial expression) categories were used from the ’manually annotated images’ subset. This subset consists of 420,299 images, indexed among two CSV files, one intended for training and one for validation. The CSV files contain a lot of information, but in this case the most important in each entry are the image path and a specific label ID. The validation file lists only 5500 entries in total, but it has a rather leveled data distribution, as opposed to the training subset consisting of 287,651 labeled images constituting eight classes varying in length, causing significant uneven data distribution. Figure 2 shows training data distribution of eight facial expressions classes within the ’manually annotated images’ subset of the AffectNet [20] dataset, clearly favoring ’happy’ and ’neutral’.

The AffectNet dataset [20] is impressive in terms of quantity and usability [20,22], however, the unleveled distribution and the huge number of samples are more of a challenge than advantage. The authors tried to limit the number of loaded samples to speed up preliminary tests, however limiting the number of samples (to 80,000) did not resolve the problem of overrepresentation of the ’happy’ class (the distribution of only the first 80,000 items is shown in the Figure 2B).

While the authors wanted to make good use of the dataset by minimizing the overrepresentation of some of the classes, this resulted in a dataset presented in Figure 1, where the samples were loaded in such a manner so that all classes would be populated equally. This approach resulted in loading all items of the less numerous classes (4, 5, 7) and a limited number of samples of the other classes (0, 1, 2, 3, 6). Some preliminary trials were performed, but a concern has arisen: will the classes 4, 5, 7 be represented within the model correctly? Will the model be able to distinguish ’disgust’ as well as it distinguishes ’happy’?

**Figure 3:**
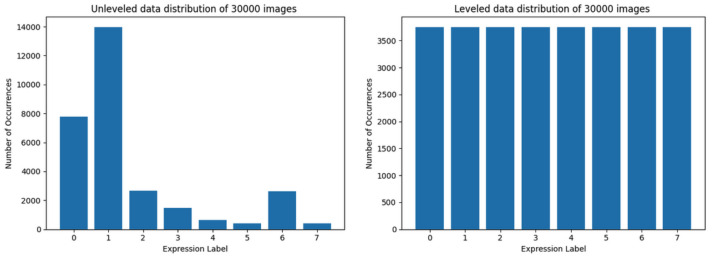
Data distribution of training set limited to 30,000 images. At the left side is data distribution using the first method, at the right—using the second method.

**Figure 4:**
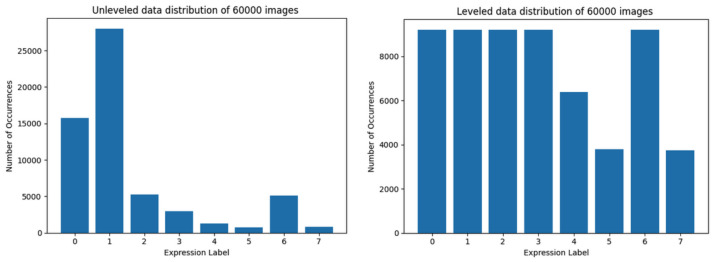
Data distribution of training set limited to 60,000 images. At the left side, the data distribution using the first method is presented, at the right—using the second method.

**Figure 5:**
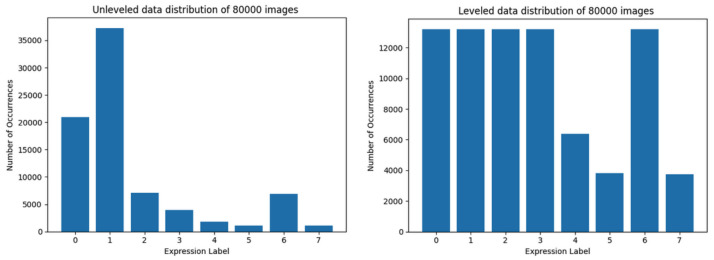
Data distribution of training set limited to 80,000 images. The left-hand side graph depicts the data distribution using the first method, while the second one—using the trimmed loading.

**Figure 6:**
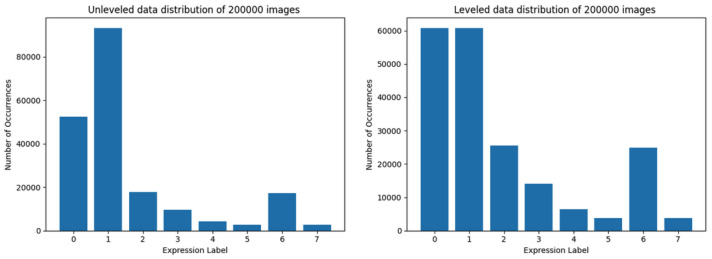
Data distribution of training set composed of 200,000 images. At the left side is data distribution using the first method, at the right using the second method.

The word ’dataset’ within this paper will be used to indicate one of the following cases:

- the whole AffectNet dataset [20],
- the training set (various versions, presented in Figure 3: left, Figure 3: right, Figure 4: left, Figure 4: right, Figure 5: left, Figure 5: right, Figure 6: left, Figure 6: right),
- the testing set (a fragment of a particular training set, subtracted from it by the DL library),
- the validation set (validation was made using a particular model after the training has finished, gives no feedback to the training).

Figure 2B shows the first 80,000 data items taken, whereas Figure 1 shows data loaded equally if possible from each category, using the above-mentioned methodology. Both figures consist of data representation of 80,000 labeled images.

### 2.1. Machine Learning Library

The size of the described part of the dataset exceeds 50 GB of raw data, thus it is necessary to load data on-the-fly or to prefetch it. The TensorFlow library contains API ’tf.data.Dataset’ [23] which provides all necessary methods and algorithms to load and preprocess data. One of mentioned methods makes it possible to decode images of different file extension formats. Images were resized to a specific resolution (which vary depending on the Neural Network used), then converted to the grayscale. To create the data structure two lists containing file paths and image labels (due to the use of Supervised Learning) are required. The dataset’s CSV file is being used for the preparation of both lists of files to be processed: (1) loading the data in the order in which it occurs in the CSV file without leveling the quantity of samples between classes, and (2) loading samples while providing the number of samples equally leveled in all classes if possible. After preparation of the initial data structure data are mapped by a method ’map’ implemented in the mentioned API. In this process the method calls another function which decodes, resizes and converts images to the grayscale, and sets a number of parallel calls. The following step is to set batch size to the same value as batch size of the Neural Network and to set prefetch buffer size. At this point it is essential to address two issues. First one is data normalization, the second one is data augmentation. The former was not implemented in the called function by the ’map’ method due to implementation of a Rescaling layer provided by Keras API, which makes sure that the values passed to the following layers are in range between 0 and 1. Application of this layer ensures consistent data normalization during training, validation and testing, thus eliminating potential interpretation mistakes. The latter one (data augmentation) was purposely not applied, because augmenting the data would certainly affect the results in a way that it could blur the differences between leveled and unleveled data to a certain degree.

### 2.2. Software and Hardware Configuration

To prepare a complete test procedure the authors have used Python in version 3.9 [24]. To load and preprocess data as well as to create and train Convolutional Neural Network (CNN) the authors have utilized several Python libraries. Data loading and preprocessing was handled by Pandas, Numpy, and ’tf.data.Dataset’ API from TensorFlow [23] library. The CNN model was created and trained by using Keras [25,26,27] implementation from the TensorFlow 2. One of the popular methods for speeding up the training of Neural Networks is by using GPU acceleration. In order to enable GPU support in the TensorFlow library it is obligatory to utilize CUDA-enabled hardware, according to the library website [28]. With this in mind, the following hardware was used during the trials: one-CPU server (Intel Xeon Silver 4208, 128 GB RAM) equipped with one GP-GPU card (NVIDIA Tesla V100 16 GB HBM2 PCIE), running Ubuntu 18.04 LTS operating system (versions 16.04 LTS and 18.04 LTS are recommended by Google/TensorFlow [28], while versions 18.04 LTS and 20.04 LTS are recommended/popular among users/community [29,30]).

### 2.3. ANN Model

Computer Vision and Image Understanding are very popular areas for research of Artificial Neural Networks (ANN), due to the possibility of visual validation of the expected results and the relative ease of explaining it and sharing the knowledge. Convolutional Neural Networks, as a type of ANNs, and as a foundation of Deep Learning, have a significant share of the Computer Vision research [26,31,32].

While the applied CNN design is not really extraordinary, and presenting it is not the goal of this paper, only the following paragraph contains its condensed description. For readers less familiar with particular terms or approaches the authors recommend the following book: [26].

As facial expression recognition is inherently a classification task [33,34,35], the output layer of the designed CNN had to be a fully connected layer with a sum of outputs equal to 1. During studies multiple of models have been designed, but the one which was utilized to provide results for this particular research was composed of a rescaling layer as input layer, followed by three blocks of layers which consisted of two each of: convolutional layers, batch normalization layers, max pooling layer and dropout layers. These blocks were followed by a flatten layer, one dense layer and finally a dense output layer with the ’Softmax’ activation function. The above mentioned blocks of layers differed from each other in layer parameters, such as number of filters in convolutional layers and dropout rates. The image input size used was 120 ×120 pixels.

### 2.4. Testing

To test the influence of data distribution the authors run multiple training sessions. The data quantities used in trainings were as follows: 30,000, 60,000, 80,000, and 200,000 faces. Every training session was performed using leveled and unleveled data for each data quantity limit. The only exception was for the largest number of training data samples, which was set at 200,000 images, which were tested only in unleveled data mode, due to the inability to trim the data set classes (due to the insufficient number of samples in the classes). Most of the trainings were completed in 50 epochs, with each training lasting approximately from 30 to over 270 min depending on the size of the training set. Training sessions utilizing a dataset of 60,000 images were performed with the number of epochs set to 50 and 80. Other training parameters, which were: initial learning rate, learning rate schedule, batch size, as well as the architecture of the CNN (including number of filters and dropout rate) have been exactly the same in order to study the influence of data quantity and data distribution. Therefore, although it is possible to perform better training sessions with fine-tuned parameter selection closer to optimal for each of the training sets individually, it would be far more challenging to objectively compare these results.

Starting from the data set size limit of 30,000 images, the distribution using both the first and the second method is shown in Figure 3. The distribution of data loaded by utilizing the second method is almost equally leveled, but the data representation (in terms of the quantity of samples required for the CNN) may be too low.

The training set of 60,000 images is the first one which can not be composed of equally leveled data in all classes (Figure 4), however the method for loading trimmed subsets of the dataset still increases data classes representation, while not increasing data distribution inequality too much.

The largest training set which consists of leveled data in classes in which data representation is presumably sufficient is composed of 80,000 images. This training set has the largest overall data representation, while it is still leveled for the majority of classes (i.e., with the exception of three classes) as shown in Figure 5.

The largest training set with the highest overall data quantity is composed of 200,000 images. Due to the lack of sufficient data representation in almost all classes (only the classes 0 and 1—’neutral’ and ’happy’—could be leveled), training ’with leveled data in most of the classes’ was impossible. The data distribution of data loaded by using method 1 and method 2 are shown in Figure 6.

Before discussing results it is important to mention that the designed Facial Expression Recognition System is using multinomial classification [26,36] (applying fully connected networks to a multiclass classification is a typical approach [26]), instead of binary classification [27,37] which would give better confidence levels due to the simplified complexity of classification (’happy’ vs ’other-than-happy’, as in one-vs-rest reduction strategy [38]). Multinomial classification (also called multiclass classification) on the other hand tries to compare every object to all classes (thus the good results may be visualized by lower values).

## 3. Results

**Figure 7:**
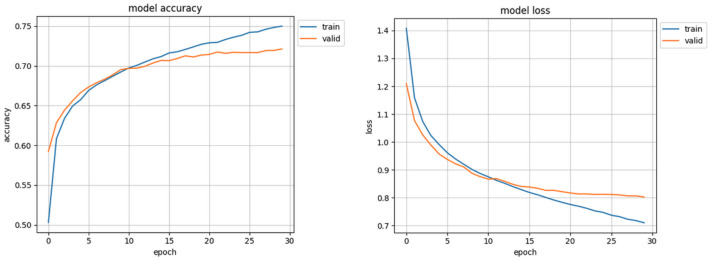
Initial training results, with incorrect validation set.

**Figure 8:**
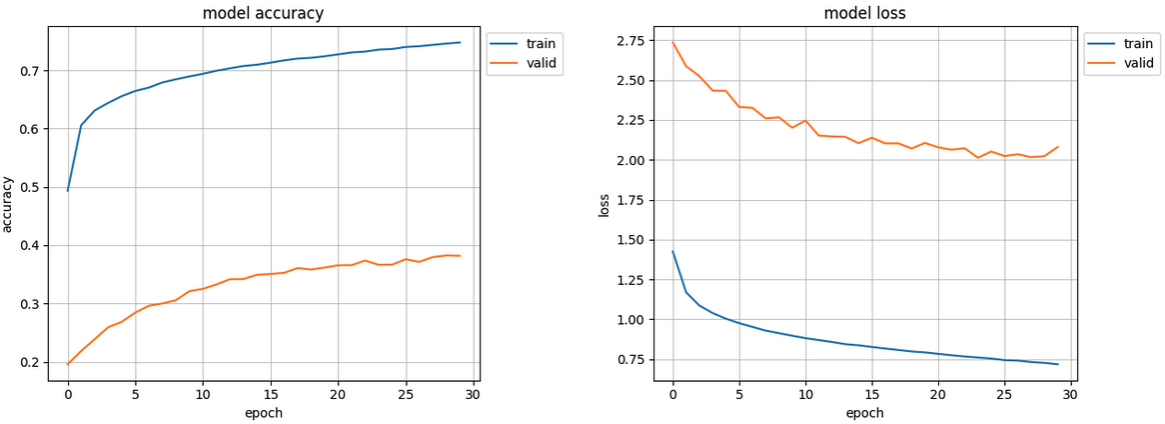
Initial training results, with correct validation set.

The initial results of the first trials of the trained model were surprising and promising: over 70% accuracy in validation and loss value of 0.80 (Figure 7) were ’too good’ especially when compared to other works [39] which also used the AffectNet dataset. In the results of detailed analysis of these trials it turned out that the Facial Expression Recognition System, designed by the authors of this study, classified almost all samples as ’happy’. Only a mere handful of faces were recognized as ’neutral’ or ’angry’, and none as ’fear’, ’disgust’ or ’sad’. It was this erroneous result that instilled in the authors the spark for searching for possible flaws of the network, conception, algorithms, and data representation. After careful examination of code a mistake has been found which resulted in not loading the correct validation set. After correcting, the model did not achieve 40% accuracy (Figure 8). That prompted authors to study the influence of data distribution on the training of the CNN model.

The results of training of the Convolutional Neural Network model using datasets depicted in Figure 3, Figure 4, Figure 5 and Figure 6 (with correct validation set, and without any method of data augmentation) are presented below, starting with the smallest training sets (Figure 3) to the largest (Figure 6). The results of the training of the model are presented side-by-side for unleveled and leveled data distribution in datasets, except for the last (largest) one.

**Figure 9:**
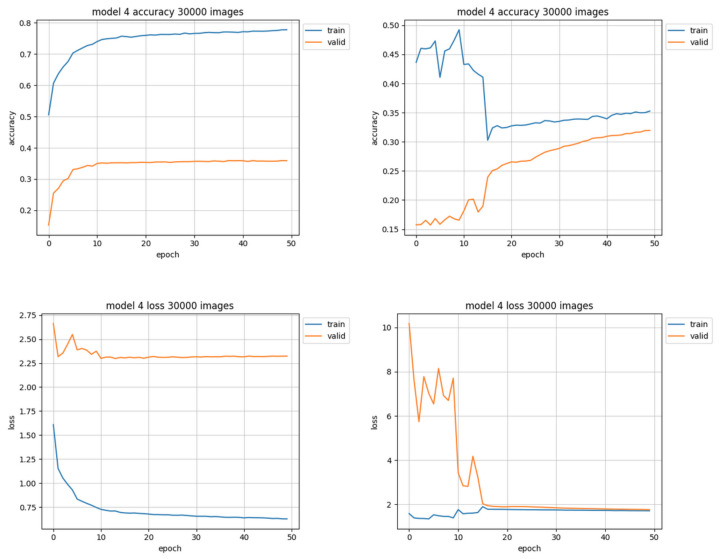
Training results using 30,000 images. Charts on the left side of the figure present results using unleveled data distribution, charts on the right side show results using leveled data distribution.

The datasets composed of 30,000 images have the most uniform distribution of objects among classes (see Figure 3, right) but 30,000 is the smallest training set used in this research—the data representation for each class in the leveled set seems to be too small for the model to learn the patterns it should be able to distinguish, as it is shown in Figure 9. When the validation curve is still at a rising trend it means that the model still can learn from presented data, but in this case the learning curve starts to flatten out. It is the only case when the accuracy of the unleveled set (Figure 9, left) appears to be better than of the leveled one (Figure 9, right), however the loss value is much higher.

**Figure 10:**
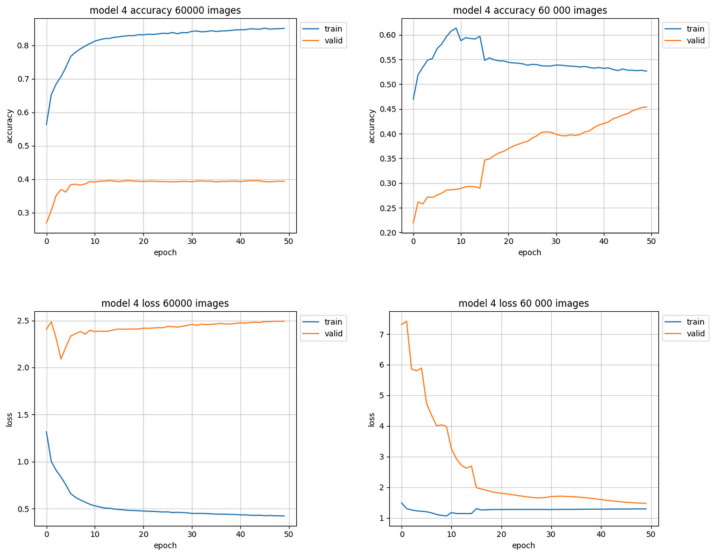
Training results using 60,000 images. Charts on the left side of figure present results using natural data distribution, charts on the right side show results using trimmed data distribution.

Training sets of 60,000 images had larger overall data representation, so they should be more useful in achieving better accuracy. However, it was also the first pair of sets in which the ’trimmed’ version could not be perfect (equally distributed) due to the quantitative underrepresentation of some of the classes. Both the equally trimmed and the natural training sets of 60,000 samples have achieved better results than those of the previous attempt (30,000). It also was the first pair of training sets to clearly show better results with trimmed data than natural in terms of accuracy. The loss value chart for trimmed data (Figure 10, right-down) is typical—it starts with a high value, but in 15 epochs drops below 2.0, reaching approximately 1.5 at the end of training. However the natural data (Figure 10, left-down) quickly loses the ability to learn from data between 5th and 10th epoch and starts to overfit—overtraining is clearly visible: the training (blue) results get better while validation (orange) results get worse.

**Figure 11:**
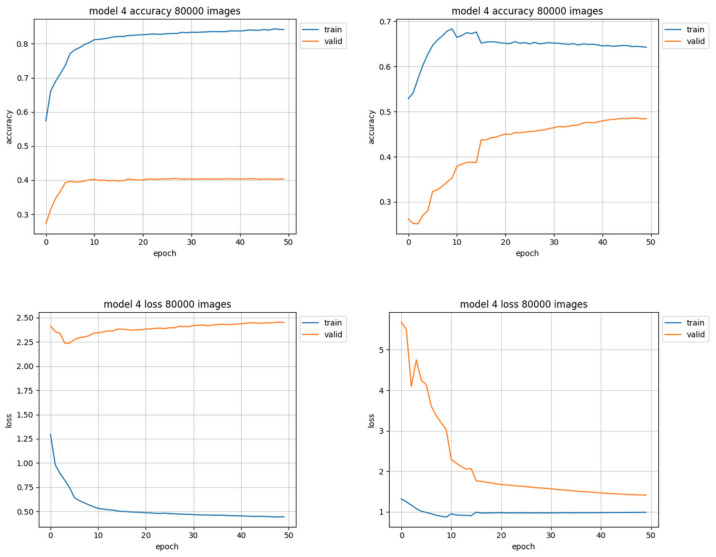
Training results using 80,000 images. Charts on the left side of figure present results using unleveled data distribution, charts on the right side show results using leveled data distribution.

The best results were achieved by the trimmed training set of 80,000 images (Figure 11, right). The unleveled data used for training the same model with the same training parameters barely did achieve a little over 40% accuracy (instead of almost 50% for leveled data), and the loss value was close to 2.5 (as opposed to 1.5 for trimmed data set). The results of this training session are shown in Figure 11.

**Figure 12:**
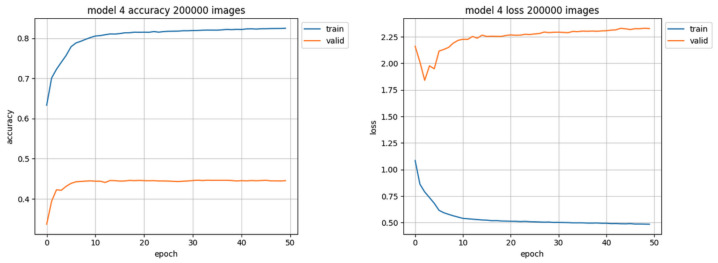
Training results using 200,000 images of the unleveled training set. The accuracy chart is presented on the left side, the loss value chart on the right side.

The largest training set, consisting of 200,000 images, has been used only on unleveled data due to the lack of sufficient data representation in almost all classes. The 200,000 training set was the only unleveled training set which resulted in model performance at the accuracy level close to 45%, but still the loss value was high (over 2.25 at the end of training) as shown in Figure 12. This training also took the longest time (exceeding 5 min for each epoch, compared to about 2 min per epoch for the set of 80,000 images), yet resulting in worse efficacy. Also the quantity of used data samples was 2.5 times larger than the best performing (second largest) training set.

## 4. Discussion

The presented results clearly show that using more leveled (trimmed) data with sufficient data representation in each class (Figure 10, right) can outperform a three times larger training set (Figure 12) in greater accuracy and lower loss value in multinomial classification. Data quality and distribution of the validation set is of no less importance than of the training set, as it is one of primary methods for checking model performance. Data distribution should always be relatively level to test if the model is capable of correct multiclass classification for all of the classes. The visible difference between the training curve and the validation curve seems to be the result of insufficient data representation of some or even most of the classes in the used dataset.

The important factor to mention is that the more numerous the data are, the more time is required to complete training. The longer one epoch takes the longer the entire training will take. This in turn means the need for more resources to process more data, and a longer training time of the model, while achieving worse results than using a smaller dataset of leveled data. This study does not indicate that having more data is worse than having less data. Having more data is indeed better, provided that the possessed data are more than less leveled, or at least that each class has a sufficient quantitative representation, while the difference between the most represented class and the rest of classes is as low as possible.

Strong disproportions between more numerous classes and less numerous may result in lowering the classification accuracy. If a specific class is quantitatively insufficient not only in the training dataset but also in the validation dataset, it is possible that the overall classification accuracy would be only slightly lower because of incorrect classification of all (scarce) samples of that class. Such multiclassification CNN model would recognize all classes with multitudinous samples and fail with most of the samples of less represented classes, numerically showing only a slight decrease in accuracy. The authors suggest that, instead of calling such a situation ’a decrease in accuracy’ (which makes it a value-oriented issue), this phenomenon should rather be called ’a stereotype’ (which makes it a cognition-oriented deficiency resulting from insufficient experience).

A very similar misclassification may occur within society, when meeting someone new—a mix of uncertainty, expectations, and experience results in specific predictions, which can obviously be wrong.

Note: the phenomenon of ’Digital Stereotypes’, considered by this paper, was observed in CNNs, however additional research should be carried out, while it may (should) also be occurring in any other Machine Learning approaches. The phenomenon relates to the dataset, not a Machine Learning model.

This research can be useful in any multiclassification applications, but especially in AI, where the classification, uncertainty and building new knowledge overlap. Something that may seem like a small dent in accuracy may eventuate the exact moment for spawning a new class of objects or a new branch of knowledge.

## 5. Conclusions

Each company aims to provide the highest customer satisfaction and attractive profit. Through a smile, it is possible to express a multitude of emotions and build new business relationships. A smile in a fraction of a second determines the credibility and openness in business and it is the smile that has the power to increase the number and affect the steadiness of the relationships built and contracts concluded. A smile expresses a positive reception in customer service, business contacts, business management and human resource management.

It should be noted that Industry 5.0, being the era of man-machine true cooperation, cognitive technologies, Machine Learning and Artificial Intelligence, the era of Big Data and Knowledge Engineering, sets new standards, challenges and expectations regarding the modern reality. Rapid changes, developments and progress will continue to shape the future of man-machine cooperation, collaboration and partnership. The current challenge is to be able to combine effectively latest advances in the fields of communication, Natural Language Processing, human cognition (including non-verbal communication), machine precision [40,41,42], to create unique products and services, as well as new quality ([43,44,45]).

The phenomenon called ’stereotype’, presented in this article, resulting from the overrepresentation of the class of objects in Machine Learning, can and should be further investigated in terms of its relationship with digital marketing, information science, business, advertising, and social computing.

## References

[1] Gardecki A., Podpora M., Beniak R., Klin B. The Pepper humanoid robot in front desk application. Proceedings of the 2018 Progress in Applied Electrical Engineering.

[2] Podpora M., Kawala-Sterniuk A., Kawala-Sterniuk A. (2019). Humanoid receptionist connected to IoT subsystems and smart infrastructure is smarter than expected. IFAC-PapersOnLine.

[3] Olaronke I., Ishaya G., Oluwaseun O., Rhoda I., Olaleke J. (2022). The Need for Robots in Global Health. Curr. J. Appl. Sci. Technol..

[4] Badzińska E. (2015). Mobilność, interaktywność i zaangażowanie użytkowników jako wyzwania współczesnej komunikacji w biznesie. Marketing i Zarządzanie.

[5] Pujari D. (2004). Self-service with a smile? Self-service technology (SST) encounters among Canadian business-to-business. Int. J. Serv. Ind. Manag..

[6] Sidikova M., Martinek R., Kawala-Sterniuk A., Ladrova M., Jaros R., Danys L., Simonik P. (2020). Vital sign monitoring in car seats based on electrocardiography, ballistocardiography and seismocardiography: A review. Sensors.

[7] Friesen E., Ekman P. (1978). Facial action coding system: A technique for the measurement of facial movement. Palo Alto.

[8] Krumhuber E., Manstead A.S. (2009). Are you joking? The moderating role of smiles in the perception of verbal statements. Cogn. Emot..

[9] Taigman Y., Yang M., Ranzato M., Wolf L. DeepFace: Closing the gap to human-level performance in face verification. Proceedings of the IEEE Computer Society Conference on Computer Vision and Pattern Recognition.

[10] Bengio Y., Lecun Y., Hinton G. (2021). Deep Learning for AI. Commun. ACM.

[11] Wu Q., Liu Y., Li Q., Jin S., Li F. The application of deep learning in computer vision. Proceedings of the 2017 Chinese Automation Congress (CAC).

[12] Stanko I., Skansi S. (2020). The Architectures of Geoffrey Hinton. Guide to Deep Learning Basics.

[13] Haselton M.G., Nettle D., Andrews P.W. (2015). The evolution of cognitive bias. The Handbook of Evolutionary Psychology.

[14] Hilbert M. (2012). Toward a synthesis of cognitive biases: How noisy information processing can bias human decision making. Psychol. Bull..

[15] Gigerenzer G. (2008). Bounded and rational. Philosophie: Grundlagen und Anwendungen/Philosophy: Foundations and Applications.

[16] MacCoun R.J. (1998). Biases in the interpretation and use of research results. Annu. Rev. Psychol..

[17] Baumgarten N., Bick E., Geyer K., Iversen D.A., Kleene A., Lindø A.V., Neitsch J., Niebuhr O., Nielsen R., Petersen E.N. (2019). Towards balance and boundaries in public discourse: Expressing and perceiving online hate speech (XPEROHS). RASK Int. J. Lang. Commun..

[18] Chen K.J., Huang C.R., Chang L.P., Hsu H.L. Sinica corpus: Design methodology for balanced corpora. Proceedings of the 11th Pacific Asia Conference on Language, Information and Computation.

[19] Caton J.N. (2020). Using linguistic corpora as a philosophical tool. Metaphilosophy.

[20] Mollahosseini A., Hasani B., Mahoor M.H. (2017). AffectNet: A New Database for Facial Expression, Valence, and Arousal Computation in the Wild. IEEE Trans. Affect. Comput..

[21] Ekman P., Friesen W.V. (1971). Constants across cultures in the face and emotion. J. Personal. Soc. Psychol..

[22] Chopra S., Hadsell R., LeCun Y. Learning a similarity metric discriminatively, with application to face verification. Proceedings of the IEEE Computer Society Conference on Computer Vision and Pattern Recognition.

[23] TensorFlow (2021). TensorFlow API Documentation–tf.data.dataset. https://www.tensorflow.org/api_docs/python/tf/data/Dataset.

[24] Python (2020). Python 3.9.0. https://www.python.org/downloads/release/python-390/.

[25] Keras (2021). About Keras. https://keras.io/.

[26] Ekman M. (2021). Learning Deep Learning: Theory and Practice of Neural Networks, Computer Vision, Natural Language Processing, and Transformers Using TensorFlow, Addison-Wesley Professional.

[27] Brownlee J. Binary Classification Tutorial with the Keras Deep Learning Library. 2016. Machine Learning Mastery. https://machinelearningmastery.com/binary-classification-tutorial-with-the-keras-deep-learning-library/.

[28] TensorFlow (2021). GPU Support. https://www.tensorflow.org/install/gpu?hl=en.

[29] Rosebrock A. (2019). Ubuntu 18.04: Install TensorFlow and Keras for Deep Learning; PyImageSearch. https://pyimagesearch.com/2019/01/30/ubuntu-18-04-install-tensorflow-and-keras-for-deep-learning/.

[30] Agarap A.F. (2020). Installing TensorFlow GPU in Ubuntu 20.04—A Short Guide for Installing TensorFlow GPU and Its Prerequisite Packages; TowardsDataScience. https://towardsdatascience.com/installing-tensorflow-gpu-in-ubuntu-20-04-4ee3ca4cb75d/.

[31] Saha S. (2018). A Comprehensive Guide to Convolutional Neural Networks—The ELI5 Way; TowardsDataScience. https://towardsdatascience.com/a-comprehensive-guide-to-convolutional-neural-networks-the-eli5-way-3bd2b1164a53/.

[32] Alzubaidi L., Zhang J., Humaidi A.J., Al-Dujaili A., Duan Y., Al-Shamma O., Santamaría J., Fadhel M.A., Al-Amidie M., Farhan L. (2021). Review of deep learning: Concepts, CNN architectures, challenges, applications, future directions. J. Big Data.

[33] Teoh K.H., Ismail R.C., Naziri S.Z.M., Hussin R., Isa M.N.M., Basir M.S.S.M. (2021). Face Recognition and Identification using Deep Learning Approach. J. Phys. Conf. Ser..

[34] Gwyn T., Roy K., Atay M. (2021). Face Recognition Using Popular Deep Net Architectures: A Brief Comparative Study. Future Internet.

[35] Yang S., Luo P., Loy C.C., Xiaoou T. (2017). Faceness-net: Face detection through deep facial part responses. IEEE Trans. Pattern Anal. Mach. Intell..

[36] Ren D., Amershi S., Lee B., Suh J., Williams J.D. (2016). Squares: Supporting interactive performance analysis for multiclass classifiers. IEEE Trans. Vis. Comput. Graph..

[37] Parker C. An analysis of performance measures for binary classifiers. Proceedings of the IEEE 11th Int. Conf. on Data Mining.

[38] Bishop C.M. (2006). Pattern Recognition and Machine Learning.

[39] Guo Y., Xia Y., Wang J., Yu H., Chen R. (2020). Real-time facial affective computing on mobile devices. Sensors.

[40] Zatwarnicki K., Zatwarnicka A. (2019). A comparison of request distribution strategies used in one and two layer architectures of web cloud systems. Computer Networks.

[41] Bender E.M., Gebru T., McMillan-Major A., Shmitchell S. On the Dangers of Stochastic Parrots: Can Language Models Be Too Big?. Proceedings of the 2021 ACM Conference on Fairness, Accountability, and Transparency.

[42] Mavridis N. (2015). A review of verbal and non-verbal human–robot interactive communication. Robot. Auton. Syst..

[43] Maddikunta P.K.R., Pham Q.V., Prabadevi B., Deepa N., Dev K., Gadekallu T.R., Ruby R., Liyanage M. (2021). Industry 5.0: A survey on enabling technologies and potential applications. J. Ind. Inf. Integr..

[44] Bryniarska A. Autodiagnosis of information retrieval on the web as a simulation of selected processes of consciousness in the human brain. Proceedings of the International Scientific Conference BCI 2018.

[45] Rudnik K., Walaszek-Babiszewska A. (2012). Probabilistic-fuzzy knowledge-based system for managerial applications. Manag. Prod. Eng. Rev..

